# High- and Reproducible-Performance Graphene/II-VI Semiconductor Film Hybrid Photodetectors

**DOI:** 10.1038/srep28943

**Published:** 2016-06-28

**Authors:** Fan Huang, Feixiang Jia, Caoyuan Cai, Zhihao Xu, Congjun Wu, Yang Ma, Guangtao Fei, Min Wang

**Affiliations:** 1 School of Materials Science and Engineering, Hefei University of Technology, Tunxi Road 193, Hefei, 230009, People’s Republic of China; 2 Key Laboratory of Materials Physics and Anhui Key Laboratory of Nanomaterials and Nanostructures, Institute of Solid State Physics, Hefei Institutes of Physical Science, Chinese Academy of Sciences, P. O. Box 1129, Hefei, 230031, People’s Republic of China

## Abstract

High- and reproducible-performance photodetectors are critical to the development of many technologies, which mainly include one-dimensional (1D) nanostructure based and film based photodetectors. The former suffer from a huge performance variation because the performance is quite sensitive to the synthesis microenvironment of 1D nanostructure. Herein, we show that the graphene/semiconductor film hybrid photodetectors not only possess a high performance but also have a reproducible performance. As a demo, the as-produced graphene/ZnS film hybrid photodetector shows a high responsivity of 1.7 × 10^7^ A/W and a fast response speed of 50 ms, and shows a highly reproducible performance, in terms of narrow distribution of photocurrent (38–65 μA) and response speed (40–60 ms) for 20 devices. Graphene/ZnSe film and graphene/CdSe film hybrid photodetectors fabricated by this method also show a high and reproducible performance. The general method is compatible with the conventional planar process, and would be easily standardized and thus pay a way for the photodetector applications.

High- and reproducible-performance photodetectors are critical to the development of many technologies, including communications, sensing, environmental protection, and imaging, etc^1^,^2^,^3^,^4^. In terms of the dimensionality of photoactive material, there exist one-dimensional (1D) nanostructure based and film based photodetectors. 1D nanostructure based photodetectors exhibit a relatively low responsivity and response speed, which is probably resulted from the low carrier mobility of intrinsic semiconductor^5^,^6^,^7^,^8^,^9^,^10^,^11^. Doping is expected to be an effective way to enhance the responsivity of 1D nanostructure photodetectors^12^,^13^,^14^, since doping is widely used to increase the carrier mobility of semiconductors. Growing 1D hybrid nanostructures with type-II band alignment is an alternative way^15^,^16^,^17^, which effectively prolongs the lifetime of photo-generated electrons and holes through forming a charge separation state. So far, most efforts only focus on the improvement of the photodetector performance, while the performance reproducibility is a common issue for single 1D nanostructure based photodetectors, especially for their future applications. Normally, the photodetector performance highly relies on the specific synthesis environment or condition of 1D nanostructure. The variation of photocurrent and response speed for a single 1D nanostructure based photodetector consisting of the same photoactive material could be up to 4 orders of magnitude^18^. Furthermore, even the nanostructures synthesized in one time experiment also demonstrate different performance because of the varied microenvironment. The photodetector performance of chlorine-doped n-type ZnS nanobelts grown from a one time experiment showed 1 order of magnitude variation^12^. Obviously, it seems very difficult or even impossible to realize both high and reproducible performance for 1D nanostructure based photodetectors.

We now consider the film based photodetectors. They possess a low performance due to low carrier mobility of the film consisting of nanocrystals. The performance has been greatly enhanced by hybridizing semiconductor films with graphene, a two-dimensional single-layer of sp^2^ bonded carbon atoms with ultrahigh carrier mobility^19^,^20^,^21^,^22^,^23^,^24^,^25^,^26^,^27^,^28^, which can provide an ultrafast transport channel for the photo-generated carriers of photoactive materials. Konstantatos *et al*. demonstrated a responsivity of 10^7^ A/W in a hybrid photodetector that consists of graphene covered with a thin film of PbS quantum dots^21^. Such a remarkable performance clearly shows the superiority of this approach. Moreover, the film based photodetector performance is collective one of all the nanocrystals composing the film, which is not so sensitive to the microenvironment of every single nanocrystal unlike a single 1D nanostructure. Thus, film based photodetectors can be reasonably assumed to have a more reproducible performance in contrast to single 1D nanostructure based photodetectors. Therefore, it is possible to realize high- and reproducible-performance photodetectors by fabricating graphene/semiconductor film hybrid photodetectors.

Herein, we show that the graphene/semiconductor film hybrid photodetectors not only possess a high performance but also have a reproducible performance, taking ZnS, ZnSe and CdSe as examples. Semiconductor films were deposited onto SiO_2_/Si substrate by high vacuum method and are supposed to have a fixed amount of photo-generated carriers at different areas. The CVD grown graphene films on Cu foil were subsequently transferred onto semiconductor film^29^,^30^,^31^, with removing PMMA carrier by low pressure annealing method, which generates a continuous graphene films and a clean semiconductor-graphene interface. Semiconductor films with a clean semiconductor-graphene interface produce a fixed amount of photo-generated carriers transferred from semiconductor to graphene. Meanwhile, continuous graphene films with uniform conductivity, confirmed by our previous work^32^, enable the photo-generated carriers to drift toward the external electrodes with almost the same transport speed. As a result, the combination of ZnS film and graphene with high carrier mobility (900 cm^2^ V^−1^ s^−1^, Supplementary Fig. S2a) achieves a high responsivity of 1.7 × 10^7^ A/W and a fast response speed of 50 ms, and shows a highly reproducible performance, in terms of narrow distribution of photocurrent (38–65 μA) and response speed (40–60 ms) for 20 devices. Graphene/ZnSe film and graphene/CdSe film hybrid photodetectors fabricated by this method also show a high and reproducible performance. Remarkably, our general method is compatible with the conventional planar process and will be easily standardized.

## Results and Discussions

**Figure 1:**
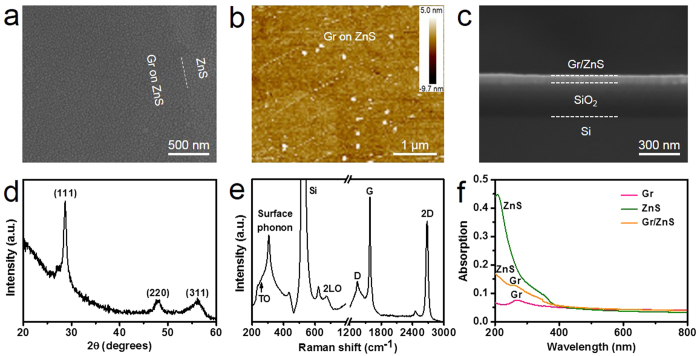
Characterization of graphene/ZnS film hybrid structures. (**a**) SEM and (**b**) AFM image of graphene/ZnS film hybrid structures. The contrast between graphene and ZnS shows the successful transfer of graphene onto ZnS film. The surface roughness of graphene/ZnS is about 1 nm, excluding some nanoparticles resulted from the transfer process (Supplementary Fig. S1). (**c**) Cross-sectional SEM image of graphene/ZnS film hybrid structures on 300 nm SiO_2_/Si substrate. The thickness of ZnS film is observed to be 60 nm, in agreement with the setting value during e-beam evaporation process. (**d**) XRD of graphene/ZnS film hybrid structures shows that ZnS is of zinc blende structure. (**e**) Raman spectrum of graphene/ZnS film hybrid structures. The peaks around 267, 305 and 674 cm^−1^ originate from the TO, surface phonon and 2LO modes of ZnS, respectively. The visible characteristic peaks of 1340, 1593 and 2693 cm^−1^ correspond to graphene D, G and 2D bands, respectively. (**f**) UV–vis absorption of graphene/ZnS film hybrid structures. The absorption peak around 267 nm of graphene originates from π plasmon peak, the absorption edge of 330 nm corresponds to ZnS band gap.

Figure 1a shows a scanning electron microscopy (SEM) image of the graphene/ZnS film hybrid composites. At the graphene edge, the contrast between graphene and ZnS under the electron irradiation indicates that the continuous graphene film has been successfully transferred onto ZnS film^31^. The surface roughness of graphene/ZnS is estimated to be about 1 nm, excluding some nanoparticles resulted from the transfer process (Fig. 1b and Supplementary Fig. S1). Cross-sectional SEM image of graphene/ZnS film hybrid structures on 300 nm SiO_2_/Si substrate shows that the thickness of ZnS film is observed to be 60 nm, in agreement with the setting value during e-beam evaporation process (Fig. 1c). The x-ray diffraction (XRD) pattern in Fig. 1d clearly reveals that the as-deposited ZnS is of zinc blende structure. The Raman spectrum is depicted in Fig. 1e. The peaks around 267, 305 and 674 cm^−1^ originate from the TO, surface phonon and 2LO modes of ZnS, respectively^33^. The visible characteristic peaks of 1340, 1593 and 2693 cm^−1^ correspond to graphene D, G and 2D bands, respectively^30^,^31^,^32^. So the appearance of both ZnS and graphene characteristic peaks in Raman spectrum shows the combination of ZnS film and graphene. To further confirm the successful transfer of graphene onto ZnS film, UV–vis absorption measurements were conducted, as shown in Fig. 1f. The absorption peak around 267 nm of graphene originates from π plasmon peak^34^, the absorption edge of 330 nm corresponds to ZnS band gap^35^,^36^, and thus the simultaneously observed graphene absorption peak and ZnS absorption edge clearly and further show the coexistence of ZnS and graphene.

**Figure 2:**
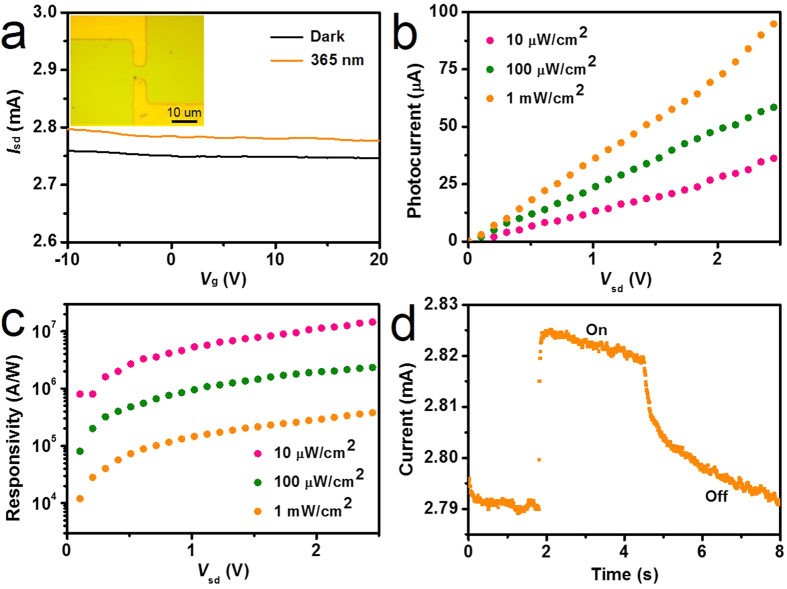
Behavior characterization of graphene/ZnS film hybrid photodetector. (**a**) Transfer characteristics as a function of back-gate voltage (*I*_sd_~*V*_g_) of graphene transistors with the underneath ZnS film under source-drain voltage *V*_sd_ = 1 V before and after 365 nm light illumination with power of 1 mW/cm^2^. The carrier transport is hole-dominated from *V*_g_ = −20 V to *V*_g_ = 20 V without the appearance of Dirac point, indicating the graphene is p-doped on ZnS film under ambience. Inset: OM image of a graphene/ZnS film hybrid photodetector. (**b**) Photocurrent and (**c**) responsivity of the graphene/ZnS film hybrid photodetector for different light powers as a function of *V*_sd_ with *V*_g_ = 0 V. (**d**) Time-dependent response of the photodetector by switching light illumination with power of 1 mW/cm^2^ on and off at *V*_sd_ = 1 V with *V*_g_ = 0 V.

To fabricate graphene/ZnS film based field effect transistors, 10/50 nm Cr/Au electrodes were deposited by thermal evaporation method with standard photolithography and lift-off processes. After 365 nm light was focused on the channel with size of 5 × 5 μm (inset in Fig. 2a), the photoconductive properties of graphene/ZnS film hybrids could be investigated (Supplementary Fig. S3a). Typical transfer characteristics of back-gated transistors for graphene transferred onto ZnS film show a hole-dominated transport without the appearance of Dirac point. This probably arises from the p-doping of oxygen in air^21^,^26^,^31^. Upon 365 nm light irradiation, the source-drain current (*I*_sd_) obviously increased regardless of the gate bias (*V*_g_) (Fig. 2a). The spectra responsivity (*R*_λ_) is a key parameter to evaluate the photodetector performance^37^, which is defined as the photocurrent generated per unit power of incident light on the effective area of a photoconductor and can be expressed as *R*_λ_ = ∆*I*/*PS*^38^. Wherein, ∆*I* is the difference between the current under illumination and dark current, namely photocurrent, *P* is the light power density irradiated on the channel, and *S* is the area of channel. Figure 2b,c display the photocurrent and the responsivity of the device as a function of the source-drain voltage (*V*_sd_) under different light power, with the back gate of *V*_g_ = 0 V. The photocurrent increases with the increase of *V*_sd_ and light power, while the calculated responsivity increases with the increase of *V*_sd_ yet decreases with the increase of the light power. It is consistent with the photodetector behavior reported before^21^,^26^. The calculated *R*_λ_ value of our fabricated graphene/ZnS film photodetector is up to 1.7 × 10^7^ A/W at an incident power of about 10 μW/cm^2^, indicating a high responsivity for our graphene/ZnS film hybrid photodetector. The responsivity can be expected to be further enhanced under light illumination with a lower power^21^. The response speed is another important parameter to assess the performance of a photodetector. The rise time and decay time are estimated to be 50 ms and 2.6 s, respectively (Fig. 2d). For comparison, the parameters about the ZnS based photodetector performance are summarized in Table 1. In contrast to single ZnS nanobelt, the graphene/ZnS film hybrid has an 8 orders of magnitude higher responsivity and a 2 orders of magnitude faster response speed^5^. Compared with the n-type doped ZnS nanobelts, the graphene/ZnS film hybrid has a comparable responsivity, but with a 3–4 orders of magnitude faster response speed^12^,^13^. In comparison with type-II ZnS based hybrids, the graphene/ZnS film hybrid has a 2–3 orders of magnitude higher responsivity and a 1–2 orders of magnitude faster response speed^15^,^16^,^17^. In contrast to the solution-grown ZnS nanobelt/graphene sandwich, the graphene/high vacuum deposited ZnS film hybrid has a 4 orders of magnitude higher responsivity and a 2 orders of magnitude faster response speed^39^, which are ascribed to the larger contact area between graphene and dense ZnS film with more absorbing light and cleaner ZnS-graphene interface, respectively. Based on the above comparison, we are able to conclude that our graphene/ZnS film hybrid photodetector has a top-level comprehensive performance.

**Figure 3:**
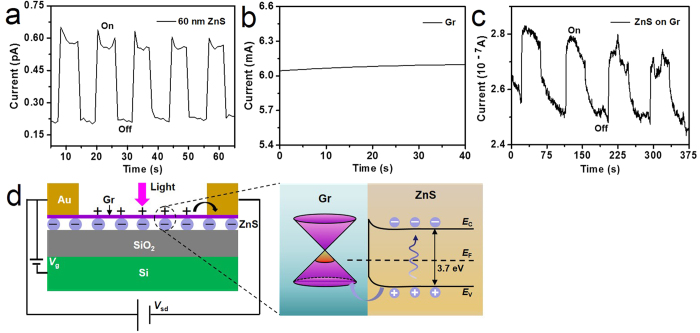
Mechanism study of graphene/ZnS film hybrid photodetector. (**a**) The time dependent response for pure ZnS film based transistor showing a response to with a photocurrent of 0.45 pA and a response speed of 2 s. (**b**) The time dependent response for pure graphene-based transistor, showing no response to 365 nm light. (**c**) The time dependent response for the detector consisting of ZnS film deposited on graphene with mobility degradation. The photocurrent is 0.02 μA with a response speed of 1.5 s. (**d**) The scheme for the proposed working mechanism of graphene/ZnS film hybrid photodetector.

The contrast experiments were designed to understand the working mechanism of the high-performance photodetectors. First of all, the photoconductive behavior of pure ZnS film and pure graphene based transistors was studied. ZnS film based transistor shows a response, but the photocurrent is only 0.45 pA with a response time of 2.0 s (Fig. 3a). Graphene-based transistor with current of mA level shows no response to 365 nm light (Fig. 3b). Photocarrier generation in graphene films is not expected to yield photoconductance due to the ultrafast recombination in graphene^40^. Subsequently, we checked the influence of graphene mobility. It was reported that graphene was seriously destroyed by physical damage during the process of deposition of Au on graphene by e-beam evaporation and the mobility would degrade sharply^41^. Thus a ZnS film/graphene hybrid detector was fabricated by means of depositing dense ZnS film on graphene. In this case, the graphene hole mobility was measured to be only 4.8 cm^2^ V^−1^ s^−1^ (Supplementary Fig. S2b), 2 orders of magnitude lower than 900 cm^2^ V^−1^ s^−1^ of normal graphene (Supplementary Fig. S2a). The produced detector exhibits a photocurrent of 0.02 μA and a response speed of 1.5 s (Fig. 3c). That is, the photocurrent and response speed strongly rely on the carrier mobility of graphene. Therefore, we attribute the high photocurrent, namely high responsivity, and fast response speed of hybrid detectors to the combination of ZnS film acting as photoactive material and graphene with a high mobility providing a fast channel. In Fig. 2a, the increase of hole-dominated current in graphene channel means that the holes transfer from ZnS film to graphene^21^,^26^,^27^. Therefore, we give the following working mechanism for a graphene/ZnS film hybrid photodetector, as shown in Fig. 3d. Upon absorption of the light with energy near or larger than the ZnS bandgap of 3.7 eV, the electron-hole pairs are generated in photoactive ZnS films. The photo-generated holes in ZnS valence band spontaneously transfer to graphene channels, forming a built-in field to equilibrate the Fermi levels. Electrons remain trapped with a typical timescale of *τ*_lifetime_ in ZnS nanocrystals as a result of the built-in field at the ZnS-graphene interface^21^. The ultrahigh carrier mobility of graphene enables large proportional holes drift to the electrodes at an ultrafast speed and thereby extremely enhances the generation of the photocurrent and the response speed.

**Figure 4:**
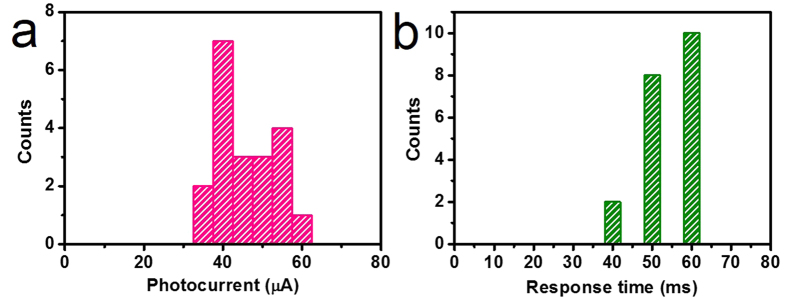
Reproducibility investigation of graphene/ZnS film hybrid photodetector. Statistics distribution for (**a**) photocurrent and (**b**) response speed of graphene/ZnS film hybrid photodetector. All photocurrent and response speed values range in 38–65 μA and 40–60 ms, respectively, showing a narrow performance distribution.

We now turn to the reproducibility investigation of the photoconductive behavior for the graphene/ZnS film hybrids. 20 photodetectors were fabricated with shadow mask to make the statistics. The time dependent response results labelled with photocurrent and response speed of 20 devices, under *V*_sd_ = 1 V with *V*_g_ = 0 V, are shown in Supplementary Fig. S5. The corresponding photocurrent and response speed statistics results in Fig. 4 demonstrate that all the photocurrent and response speed values range in 38–65 μA and 40–60 ms, respectively, showing a narrow distribution. The performance variation of a single nanostructure based photodetector consisting of the same photoactive material could be up to 4 orders of magnitude^18^. The conductivity of chlorine-doped n-type ZnS nanobelts grown from a one time experiment ranged from 1 to 10 Scm^−1^, showing 1 order of magnitude variation, and the photodetector performance consequently appeared 1 order of magnitude difference^12^. The existed variation for the performance of ZnS nanobelt based photodetectors is because of the individual difference of ZnS nanobelt resulted from the varied synthesis microenvironment. Compared with the results, our photodetector performance shows an excellent reproducibility. In our work, ZnS films in 20 different channels are supposed to have a fixed amount of photo-generated carriers. The annealing method for removing PMMA carrier under low pressure is supposed to produce continuous graphene films and to generate a clean semiconductor-graphene interface. Semiconductor films with a clean semiconductor-graphene interface produce a fixed amount of photo-generated carriers transferred from semiconductor to graphene. Meanwhile, continuous graphene films with uniform conductivity, shown by our previous work^32^, enable the photo-generated carriers transferred from semiconductor films to drift toward the external electrodes with almost the same transport speed. It is confirmed by the narrow distribution of the dark current (5.95–11.85 mA) of 20 graphene/ZnS film hybrid photodetectors, shown in Supplementary Fig. S5. The above-mentioned points make graphene/ZnS film hybrid photodetectors have a narrow distribution of photocurrent and response speed.

**Figure 5:**
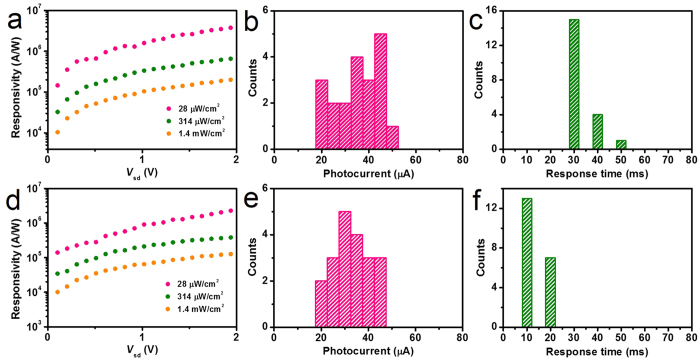
High- and reproducible-performance graphene/ZnSe film and graphene/CdSe film photodetector. Responsivity of (**a**) graphene/ZnSe film hybrid photodetector and (**d**) graphene/CdSe film hybrid photodetector for different light powers as a function of *V*_sd_ with *V*_g_ = 0 V with light wavelength of 460 nm and 620 nm, respectively. Photocurrent statistics distribution for (**b**) graphene/ZnSe film and (**e**) graphene/CdSe film hybrid photodetector. Response speed statistics distribution for (**c**) graphene/ZnSe film and (**f**) graphene/CdSe film hybrid photodetector. All photocurrent and response speed values range in 22–54 μA and 30–50 ms for graphene/ZnSe film device and 22–50 μA and 10–20 ms for graphene/CdSe film device, respectively, showing a narrow performance distribution.

The developed method was applied to other graphene/semiconductor film hybrid photodetector fabrication, using ZnSe and CdSe as examples. The photoconductive behavior of graphene/ZnSe film and graphene/CdSe film hybrid photodetectors is shown in Supplementary Fig. S6. The combination of ZnSe film with graphene and the combination of CdSe film with graphene achieve a high responsivity of 3.9 × 10^6^ A/W with a fast response speed of 30 ms and a high responsivity of 2.3 × 10^6^ A/W with a fast response speed of 10 ms (Fig. 5a,d), respectively. Compared with single ZnSe nanostructure, the graphene/ZnSe film hybrids have a 5–7 orders of magnitude higher responsivity and a 1–2 orders of magnitude faster response speed^42^,^43^,^44^,^45^, as shown in Table 2. The working mechanism of graphene/ZnSe film and graphene/CdSe film hybrid photodetectors was similar to that of graphene/ZnS film hybrid photodetector (Fig. 3). They also possess a highly reproducible performance, in terms of narrow distribution of photocurrent (22–54 μA) with response speed (30–50 ms) (Fig. 5b,c) and photocurrent (22–50 μA) with response speed (10–20 ms) (Fig. 5e,f) for 20 devices, respectively. The narrow distribution of 5.05–12.53 mA for the dark current of three kind of hybrid photodetectors (60 devices) in Supplementary Figs S5, S10 and S11 confirms that the graphene films with the uniform conductivity could be obtained by our method. Note that the dark current is determined by graphene because semiconductor film has far lower current than graphene (Fig. 3a,b). The as-produced three type of devices all have narrow distribution for photocurrent and response time, suggesting that semiconductor film with fixed amount of photo-generated carriers and uniform charge transfer behavior at semiconductor-graphene interface could be guaranteed by our method via producing semiconductor film with a clean semiconductor-graphene interface. Noticeably, the extended study proves the universality of our developed method. As well, a high responsivity of 10^7^ for ligand-bridged PbS film/graphene hybrid photodetectors was reported by the other two independent groups^21^,^27^. It further indicates the potential for standardizing the production of high- and reproducible-performance graphene/semiconductor film hybrid photodetectors by all the groups, unlike that the photodetectors based on a single 1D nanostructure produced by different groups appear a huge variation up to 4 orders of magnitude^18^.

In summary, the combination of graphene films possessing an ultrahigh mobility and uniform conductivity with semiconductor films having a fixed amount of photo-generated carriers allows us to achieve graphene/semiconductor film hybrid photodetectors with a high and reproducible performance, based on that film based detector has a reproducible performance because it is collective one of the nanocrystals in film. As a demo, the produced graphene/ZnS film hybrid photodetectors shows a high responsivity of 1.7 × 10^7^ A/W and a fast response speed of 50 ms, and shows a highly reproducible performance, in terms of narrow distribution of photocurrent (38–65 μA) and response speed (40–60 ms) for 20 devices. The method universality is confirmed by the realization of graphene/ZnSe film and graphene/CdSe film hybrid photodetectors with a high and reproducible performance. Our general method developed in this work is compatible with the conventional planar process, and would be easily standardized and pay a way for the detector applications.

## Methods

### Preparation of ZnS, ZnSe and CdSe film

ZnS and ZnSe film with the thickness of 60 nm was deposited by e-beam evaporation, and 60 nm thick CdSe film was produced by thermal evaporation. ZnS, ZnSe and CdSe crystals (99.99% purity) with size of mm scale were used as targets. After the standard cleaning, the Si wafer with 300 nm SiO_2_ was utilized as substrate for the semiconductor film deposition. The vacuum remained 2 × 10^−4^ Pa during the whole deposition process. Before graphene transfer, the semiconductor film on Si/SiO_2_ substrate was annealed for 30 min at 300 °C under low pressure with 10 sccm H_2_ and 20 sccm Ar, enabling the film adhered to the substrate as it was placed in water to pick up the graphene films.

### Graphene growth and transfer

The details of graphene growth and transfer can be found in our previous work^31^,^32^, except that the PMMA carrier for graphene transfer was removed by annealing for 2 hours at 400 °C under low pressure with 10 sccm H_2_ and 20 sccm Ar. This method enables the crackles transfer of graphene.

### Transistor fabrication

After graphene was transferred onto ZnS, ZnSe and CdSe film on 300 nm SiO_2_/Si, photolithography was used to fabricate transistors with channel lengths of 5 μm and widths 5 μm. For the performance reproducibility investigation of the graphene/ZnS, graphene/ZnSe and graphene/CdSe hybrids, 20 devices with channel length of 20 μm and width of 500 μm were fabricated with shadow mask. The doped Si substrate served as a global back gate. 10/50 nm Cr/Au used as the electrical contact metal for the source and drain electrodes were deposited by a high-vacuum thermal evaporation system.

### Characterization and measurement

SEM images were taken with a Hitachi SU8020 scanning electron microscope operated at 1 kV. XRD patterns of the samples were recorded with an X-ray diffractometer (X’ Pert Pro MPD) with λ = 1.54056 Å. Raman spectroscopy was performed on the samples with a confocal microprobe Raman spectrometer (Renishaw). UV-vis absorption (UV-3600) was conducted on the samples after transfer onto quartz substrate. The electrical and photoconductive properties of the graphene/ZnS film, graphene/ZnSe film and graphene/CdSe film hybrid devices were measured by a semiconductor parameter analyzer system (Keithley 2636B) at room temperature, using light emitting diodes with the corresponding wavelength of 365 nm, 460 nm and 620 nm as the incident light.

## Additional Information

**How to cite this article**: Huang, F. *et al*. High- and Reproducible-Performance Graphene/II-VI Semiconductor Film Hybrid Photodetectors. *Sci. Rep.* **6**, 28943; doi: 10.1038/srep28943 (2016).

## Supplementary Material

Supplementary Information

## Figures and Tables

**Table 1:** Summary and comparison of the characteristic parameters of graphene/ZnS film hybrid and other ZnS based photodetectors.

Photodetectors	Dark current	Photocurrent	*R*_*λ*_ (A/W)	Rise time	Decay time	Reference
ZnS nanobelts	0.08 pA (5.0 V)	0.5 pA (5.0 V)	0.12	<1 s	<1 s	5
Cl-doped ZnS nanobelts	1 μA (1.0 V)	10 μA (1.0 V)	2.9 × 10^6^	76 s	463 s	12
Al-doped ZnS nanobelts	0.15 μA (5.0 V)	0.6 μA (5.0 V)	3 × 10^7^	450 s	900 s	13
ZnS/ZnO biaxial nanobelts	0.67 μA (5.0 V)	4.97 μA (5.0 V)	5 × 10^5^	<0.3 s	1.7 s	14
Branched ZnS-ZnO hybrids	6 pA (10.0 V)	59 pA (10.0 V)	–	0.77 s	0.73 s	15
ZnS/SnO_2_ core shell nanobelts	0.4 μA (1.0 V)	2.8 μA (1.0 V)	6.2 × 10^4^	8 s	61 s	16
ZnS nanobelt/graphene sandwich	7 μA (1.0 V)	36 μA (1.0 V)	1.9 × 10^3^	2.8 s	7.5 s	39
Graphene/ZnS film hybrids	2.79 mA (1.0 V)	35 μA (1.0 V)	1.7 × 10^7^	0.05 s	3.5 s	This work

**Table 2:** Summary and comparison of the characteristic parameters of graphene/ZnSe film hybrid and other ZnSe based photodetectors.

Photodetectors	Dark current	Photocurrent	*R*_*λ*_ (A/W)	Rise time	Decay time	Reference
ZnSe nanowires	29 nA (0.1 V)	2.7 μA (1.0 V)	–	<1 s	<1 s	42
ZnSe nanowires	180 nA (8.0 V)	–	22	–	–	43
ZnSe Nanobelts	<10^−4^ pA (30.0 V)	1.89 pA (30.0 V)	0.12	<0.3 s	<0.3 s	44
Sb doped ZnSe nanowires	4.3 nA (5.0 V)	0.26 μA (5.0 V)	–	<1 s	<1 s	45
Graphene/ZnSe film hybrids	2.43 mA (1.0 V)	40 μA (1.0 V)	3.9 × 10^6^	0.03 s	0.04 s	This work
