# Supplementary material for: High- and Reproducible-Performance Graphene/II-VI Semiconductor Film Hybrid Photodetectors
Source: Sci Rep. 2016 Jun 28;6:28943. doi: 10.1038/srep28943 (PMC4937183; doi:10.1038/srep28943)
Supplement: Supplementary Information [file srep28943-s1.doc]

Supplementary Information for

High- and Reproducible-Performance Graphene/II-VI Semiconductor Film Hybrid Photodetectors

Fan Huang^1^, Feixiang Jia^1^, Caoyuan Cai^1^, Zhihao Xu^1^, Congjun Wu^1^, Yang Ma^1^, Guangtao Fei^2^ & Min Wang^1^

^1^School of Materials Science and Engineering, Hefei University of Technology, Tunxi Road 193, Hefei, 230009, People’s Republic of China

^2^Key Laboratory of Materials Physics and Anhui Key Laboratory of Nanomaterials and Nanostructures, Institute of Solid State Physics, Hefei Institutes of Physical Science, Chinese Academy of Sciences, P. O. Box 1129, Hefei, 230031, People’s Republic of China

*Corresponding author: E-mail: minwang@hfut.edu.cn (M. Wang) gtfei@issp.ac.cn (G. Fei)

**Contents**

**1. Roughness characterization of graphene/ZnS films**

**2. Graphene transfer characteristics**

**3. Stability investigation of graphene/ZnS film photodetector**

**4. Spectroscopic photoresponse of graphene/ZnS film photodetector**

**5. Time dependent response of 20 graphene/ZnS film photodetectors**

**6. ZnS film thickness dependence for photocurrent of graphene/ZnS film photodetectors**

**7. Characterization of graphene/ZnSe (CdSe) film photodetectors**

**8. Stability investigation of graphene/ZnSe (CdSe) film photodetector**

**9. Spectroscopic photoresponse of graphene/ZnSe (CdSe) film photodetector**

**10. Time dependent response of 20 graphene/ZnSe film photodetectors**

**11. Time dependent response of 20 graphene/CdSe film photodetectors**

**12. References**

**1. Roughness characterization of graphene/ZnS films**

After graphene was transferred on ZnS films deposited on 300 nm SiO_2_/Si substrate, the roughness of graphene/ZnS films was characterized by AFM. The roughness is estimated to be around 1 nm excluding particles resulted from the transfer process, as shown in right panel in Figure S1a.

**
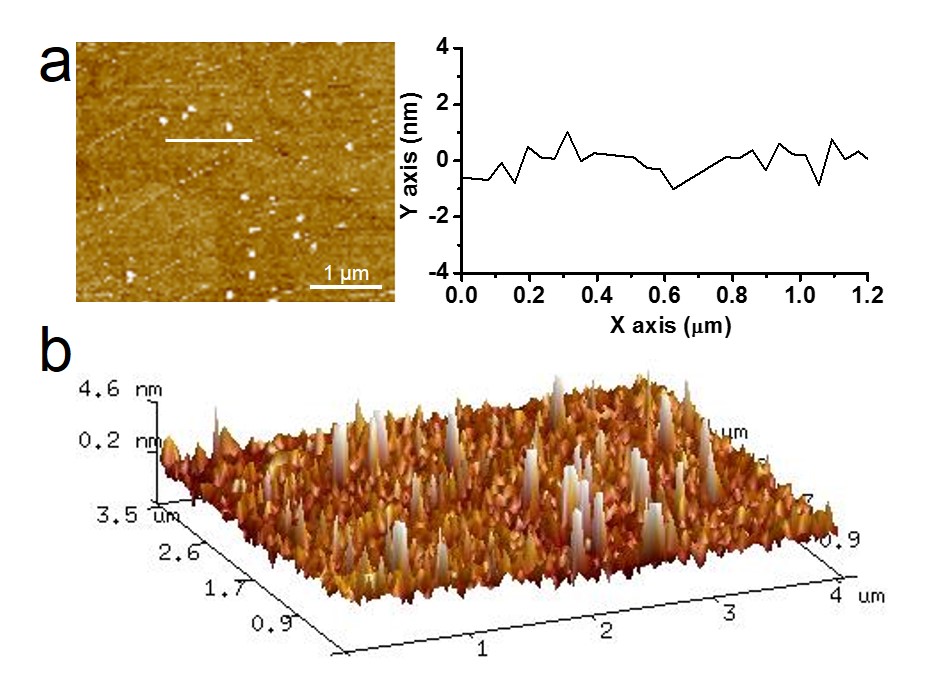
**

**Figure S1.** (**a**) Roughness characterization of graphene after transfer on ZnS films. The roughness is estimated to be around 1 nm. (**b**) AFM image of 3D view corresponding to AFM image in (**a**) with rotation of 30^o^.

**2. Graphene transfer characteristics**

Typical transfer characteristics of back-gated transistors for graphene transferred onto ZnS film and for graphene after depositing ZnS film show a hole-dominated transport without the appearance of Dirac point. This is probably caused by the p-doping of oxygen in air^1-3^. The hole mobility was calculated using the equation *μ* = dσ/(*C*_g_ ∙ d*V*_g_) = (*L*/*C*_g_*WV*_d_) ∙ (*dI*_d_/*dV*_g_), where *μ* is the field effect mobility, *C*_g_ is the gate capacitance of the SiO_2_ dielectric, and *I*_d_ is the drain current. *dI*_d_/*dV*_g_ was estimated from the slope between *V*_g_ = -10 V and *V*_g_ = 0 V. The calculated hole mobility of the graphene transferred onto ZnS film and graphene after depositing ZnS film was 900 and 4.8 cm^2^ V^-1^ s^-1^, respectively. The reduction of graphene carrier mobility was ascribed to the graphene damage during the ZnS film deposition process.

**
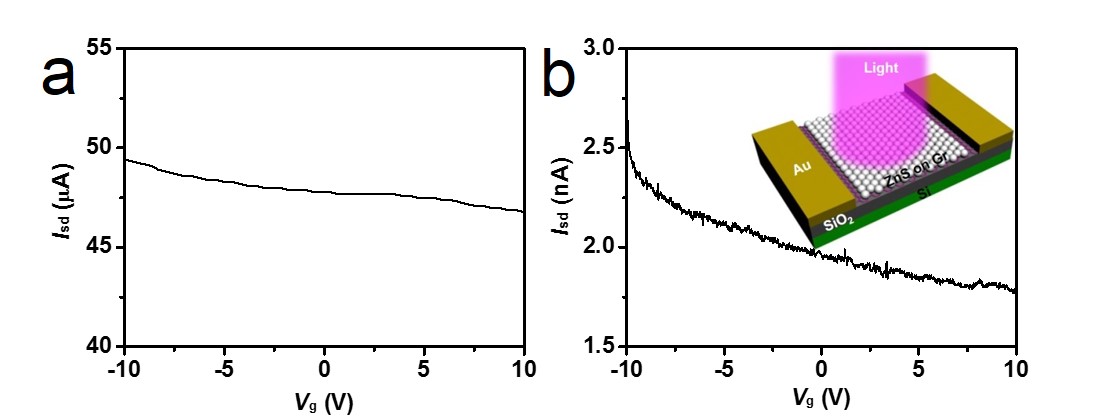
**

**Figure S2.** Typical transfer characteristics of back-gated transistors at source-drain voltage *V*_sd_ = 10 mV for (**a**) graphene transferred onto ZnS film, and (**b**) graphene after depositing ZnS film. The inset is the scheme of the graphene/ZnS film hybrid photodetector.

**3. Stability investigation of graphene/ZnS film photodetector**

The stability of graphene/ZnS film photodetector was investigated. Figure S3b depicts a time-dependent response of graphene/ZnS film device by switching light illumination on and off periodically with 5 cycles at *V*_sd_ = 1 V with *V*_g_ = 0 V. The device shows a good stability upon 365 nm illumination with power of 1 mW/cm^2^.

**
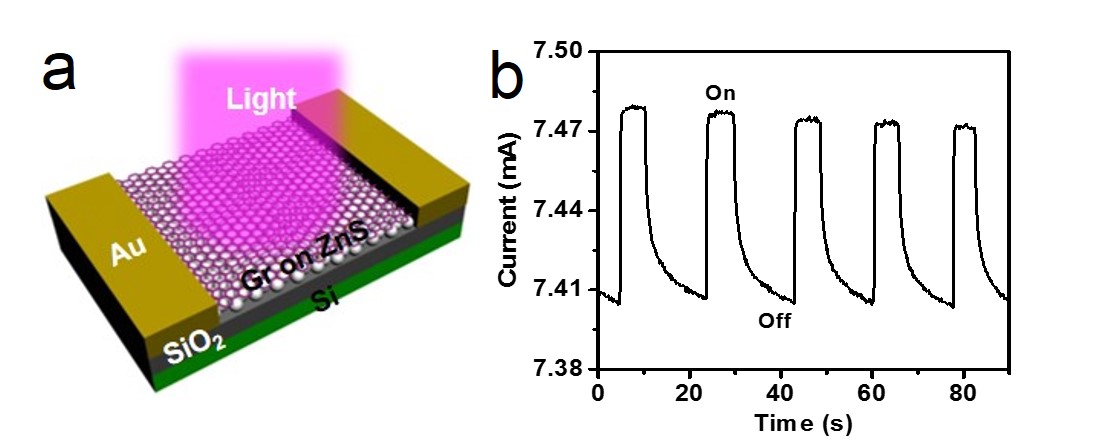
**

**Figure S3.** (**a**) Scheme of the graphene/ZnS film hybrid photodetector. (**b**) The time-dependent response of graphene/ZnS film device by switching light illumination on and off periodically with 5 cycles at *V*_sd_ = 1 V with *V*_g_ = 0 V.

**4. Spectroscopic photoresponse of graphene/ZnS film photodetector**

The cut-off edge of spectral photoresponse for graphene/ZnS film photodetector was studied. Figure S4 shows a spectroscopic photoresponse of graphene/ZnS film device as a function of light wavelength with power of 20 μW/cm^2^ at *V*_sd_ = 1 V with *V*_g_ = 0 V. There is no response at the wavelength longer than 402 nm, while the photocurrent sharply increases at the wavelength shorter than the threshold.


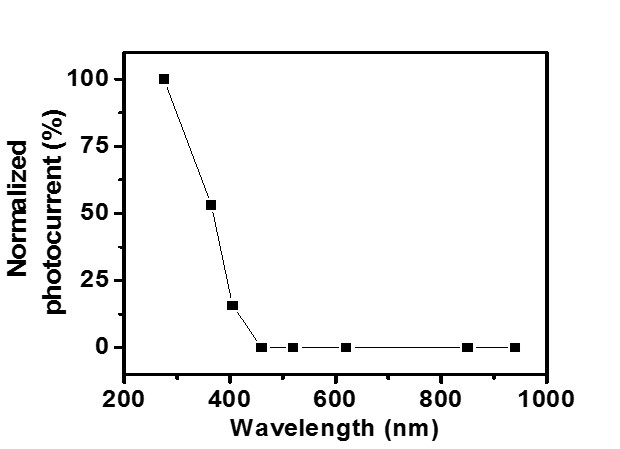


**Figure S4.** The spectroscopic photoresponse of graphene/ZnS film device as a function of the incident light wavelength at *V*_sd_ = 1 V with *V*_g_ = 0 V.

**5. Time dependent response results of 20 graphene/ZnS film photodetectors**

For the reproducibility investigation of the photoconductive performance for the graphene/ZnS film hybrids, 20 photodetectors with channel length of 20 μm and width of 500 μm were fabricated by shadow mask. This fabrication method by means of shadow mask could achieve high yield efficiency. The time dependent response curves of 20 devices at *V*_sd_ = 1 V with *V*_g_ = 0 V are shown in Figure S5.


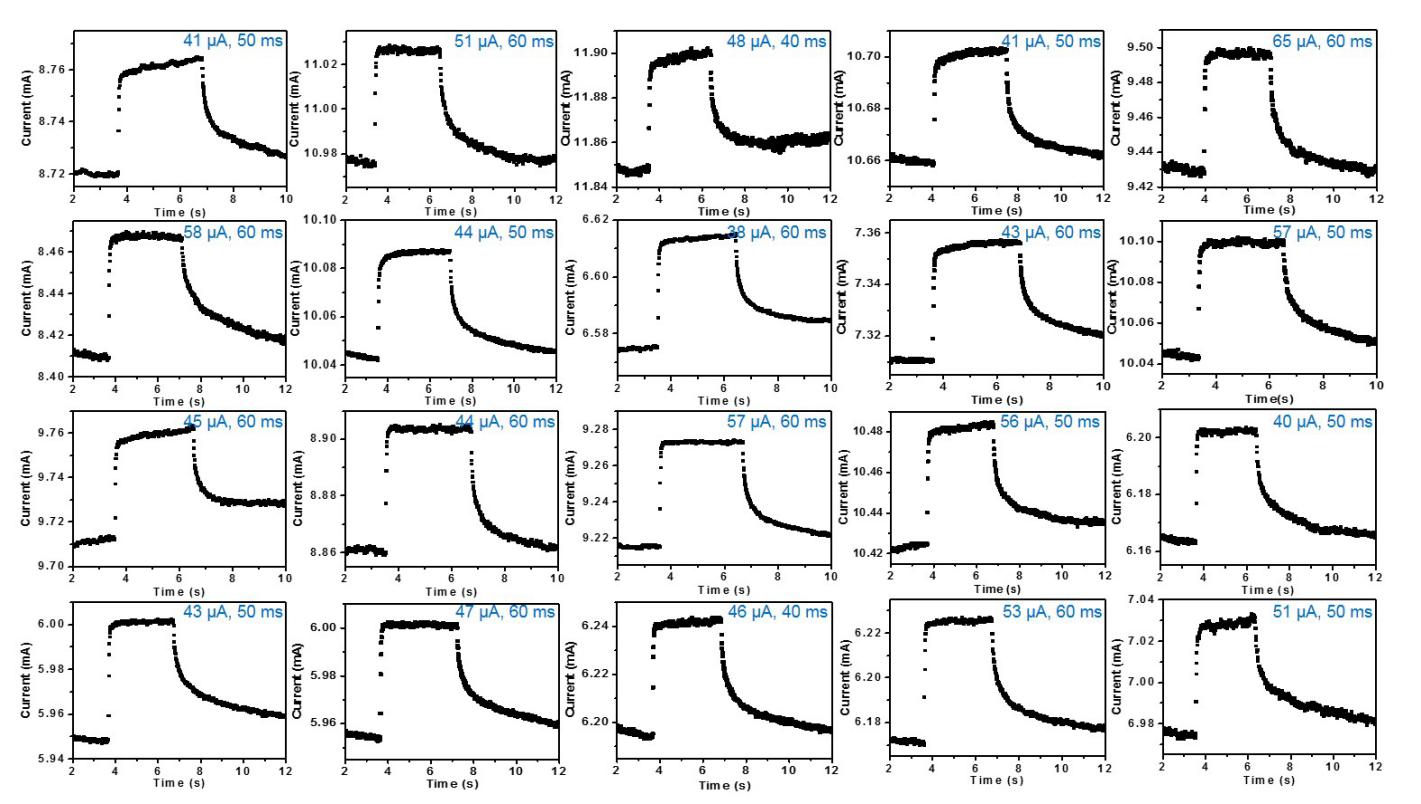


**Figure S5.** The time dependent response results of 20 devices at *V*_sd_ = 1 V with *V*_g_ = 0 V, by switching 365 nm light illumination with power of 1 mW/cm^2^ on and off.

**6. ZnS film thickness dependence for photocurrent of graphene/ZnS film photodetectors**

The ZnS film thickness dependence for photocurrent of the graphene/ZnS film hybrid photodetector was investigated, as shown in Figure S6. The photocurrent linearly increases with the increase of ZnS film thickness as the thickness is less than 60 nm. The photocurrent almost remains unchanged when the thickness increases to 90 nm, which means that the optimized thickness is 60 nm.

**
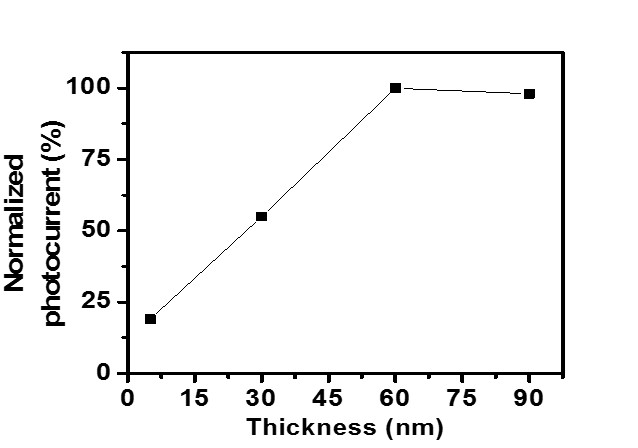
**

**Figure S6.** The normalized photocurrent as a function of ZnS film thickness.

**7. Characterization of ZnSe (CdSe) film/graphene photodetectors**

Figure S7a and S7d show the typical transfer characteristics of back-gated transistors for graphene transferred onto ZnSe and CdSe film with a hole-dominated transport, respectively. Upon 460 nm and 620 nm light irradiation, *I*_sd_ obviously increased regardless of the gate bias, as shown in Figure S7a and S7d, respectively. The results mean that the photo-generated holes in ZnSe and CdSe valence band transfer to graphene channels, and the ZnSe (CdSe) film/graphene hybrid photodetectors have the same working mechanism with graphene/ZnS film hybrid photodetector (Fig. 3). Figure S7b and S7e display the photocurrent of graphene/ZnSe film and graphene/CdSe film hybrid device as a function of *V*_sd_ under different light power with the back gate of *V*_g_ = 0 V, respectively. The photocurrent increases with the increase of *V*_sd_ and light power. It is consistent with the photodetector behavior reported before^1^. The rise time and decay time are estimated to be 30 ms and 40 ms for graphene/ZnSe film and 10 ms and 40 ms for graphene/CdSe film, respectively (Fig. S7c and S7f).


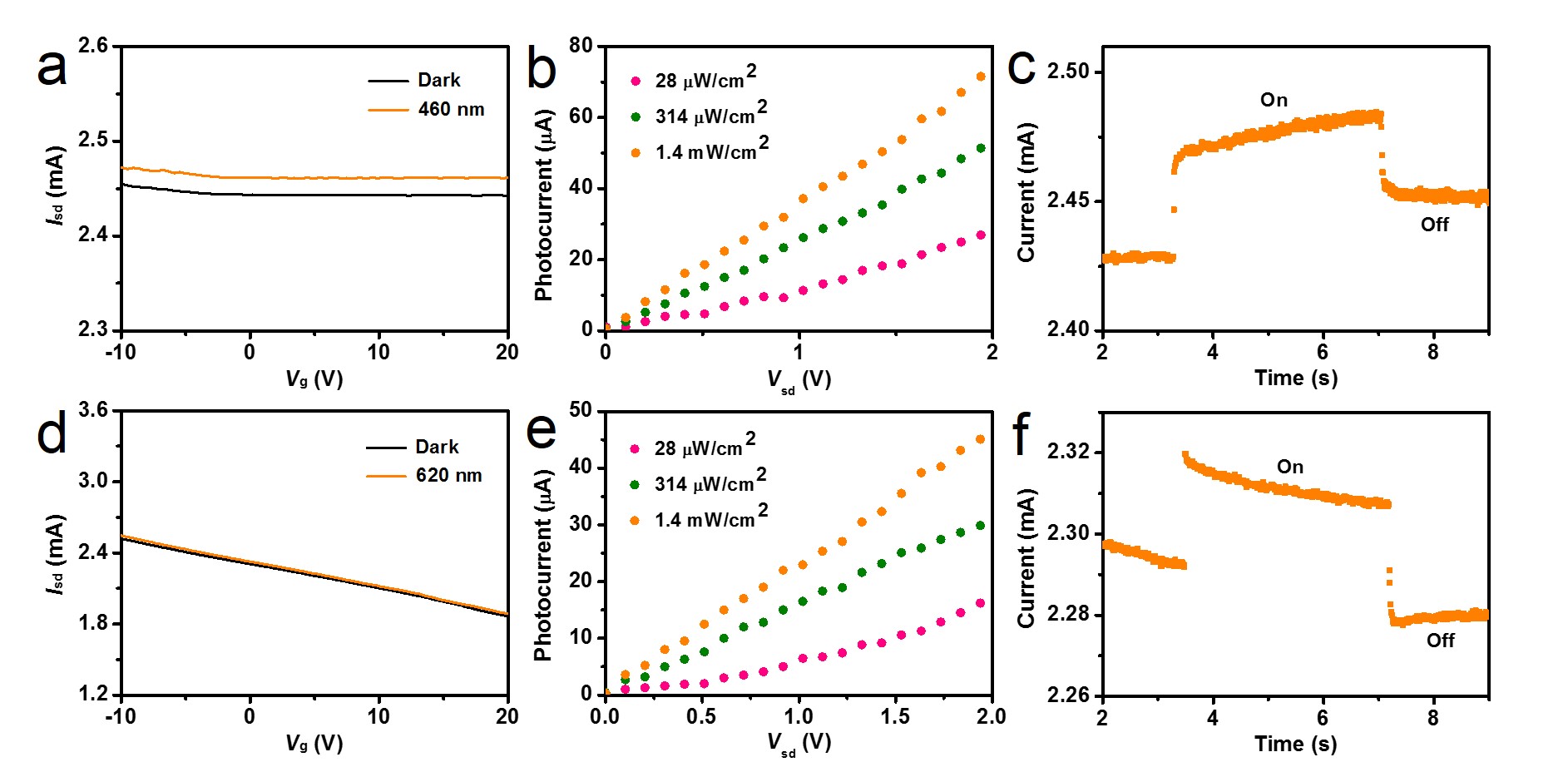


**Figure S7.** Transfer characteristics as a function of back-gate voltage (*I*_sd_ ∼ *V*_g_) of graphene transistors with the underneath (**a**) ZnSe and (**d**) CdSe film under *V*_sd_ = 1 V before and after 460 nm and 620 nm light illumination with power of 1.4 mW/cm^2^, respectively. Photocurrent and of (**b**) graphene/ZnSe film and (**e**) graphene/CdSe film hybrid photodetector for different light powers as a function of *V*_sd_ with *V*_g_ = 0 V. Time-dependent response of (**c**) graphene/ZnSe film and (**f**) graphene/CdSe film hybrid photodetector by switching light illumination with power of 1.4 mW/cm^2^ on and off at *V*_sd_ = 1 V with *V*_g_ = 0 V.

**8. Stability investigation of graphene/ZnSe (CdSe) film photodetector**

The stability of graphene/ZnSe and graphene/CdSe film photodetector was investigated. The graphene/ZnSe and graphene/CdSe film devices show a good stability by switching 460 nm and 620 nm light illumination on and off periodically with 6 and 5 cycles at *V*_sd_ = 1 V with *V*_g_ = 0 V, respectively.


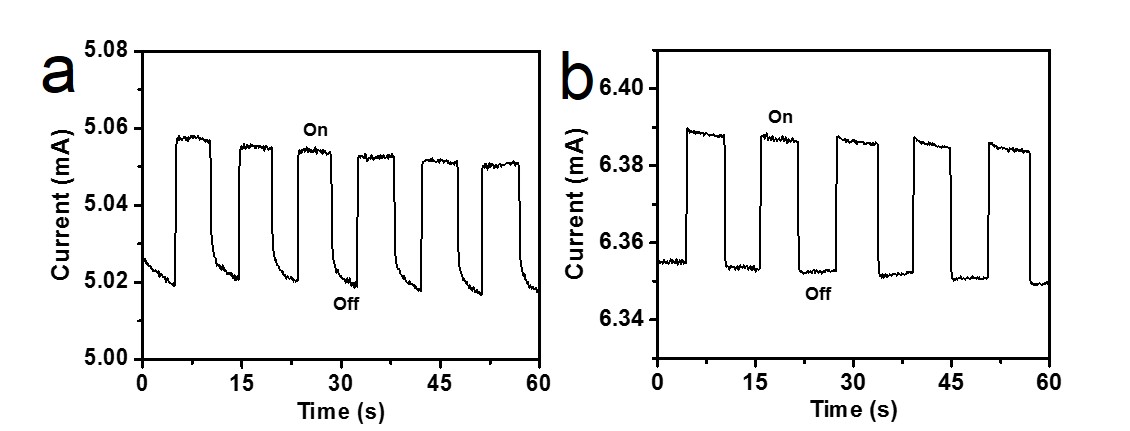


**Figure S8.** The time-dependent response of (**a**) graphene/ZnSe film and (**b**) graphene/CdSe film device by switching 460 nm and 620 nm light illumination on and off periodically at *V*_sd_ = 1 V with *V*_g_ = 0 V.

**9. Spectroscopic photoresponse of graphene/ZnSe (CdSe) film photodetector**

Figure S9 shows a spectroscopic photoresponse of graphene/ZnSe and graphene/CdSe film device as a function of light wavelength of with power of 1.4 mW/cm^2^ at *V*_sd_ = 1 V with *V*_g_ = 0 V. The responsivity of graphene/ZnSe hybrid photodetector sharply increases at the wavelength shorter than the threshold of 495 nm. The cut-off edge of graphene/CdSe hybrid photodetector is around 655 nm. The different threshold value is ascribed to the different band gap of the photoactive material.

**
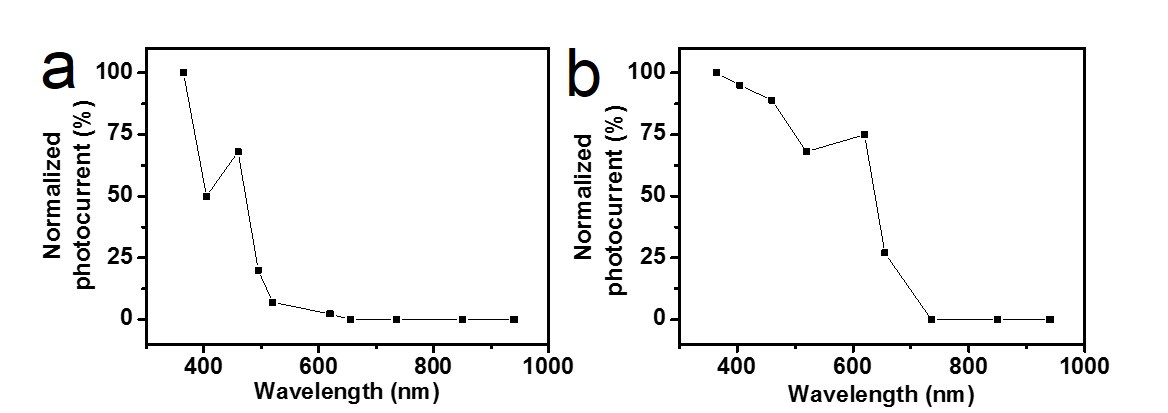
**

**Figure S9.** The spectroscopic photoresponse of (**a**) graphene/ZnSe film and (**b**) graphene/CdSe film device as a function of the incident light wavelength at *V*_sd_ = 1 V with *V*_g_ = 0 V.

**10. Time dependent response results of 20 graphene/ZnSe film photodetectors**

For the reproducibility investigation of the photoconductive performance for the graphene/ZnSe film hybrids, 20 photodetectors with channel length of 20 μm and width of 500 μm were fabricated by shadow mask. The time dependent response curves of 20 devices at *V*_sd_ = 1 V with *V*_g_ = 0 V are shown in Figure S10, with the values of photocurrent and response speed labelled in each figure.


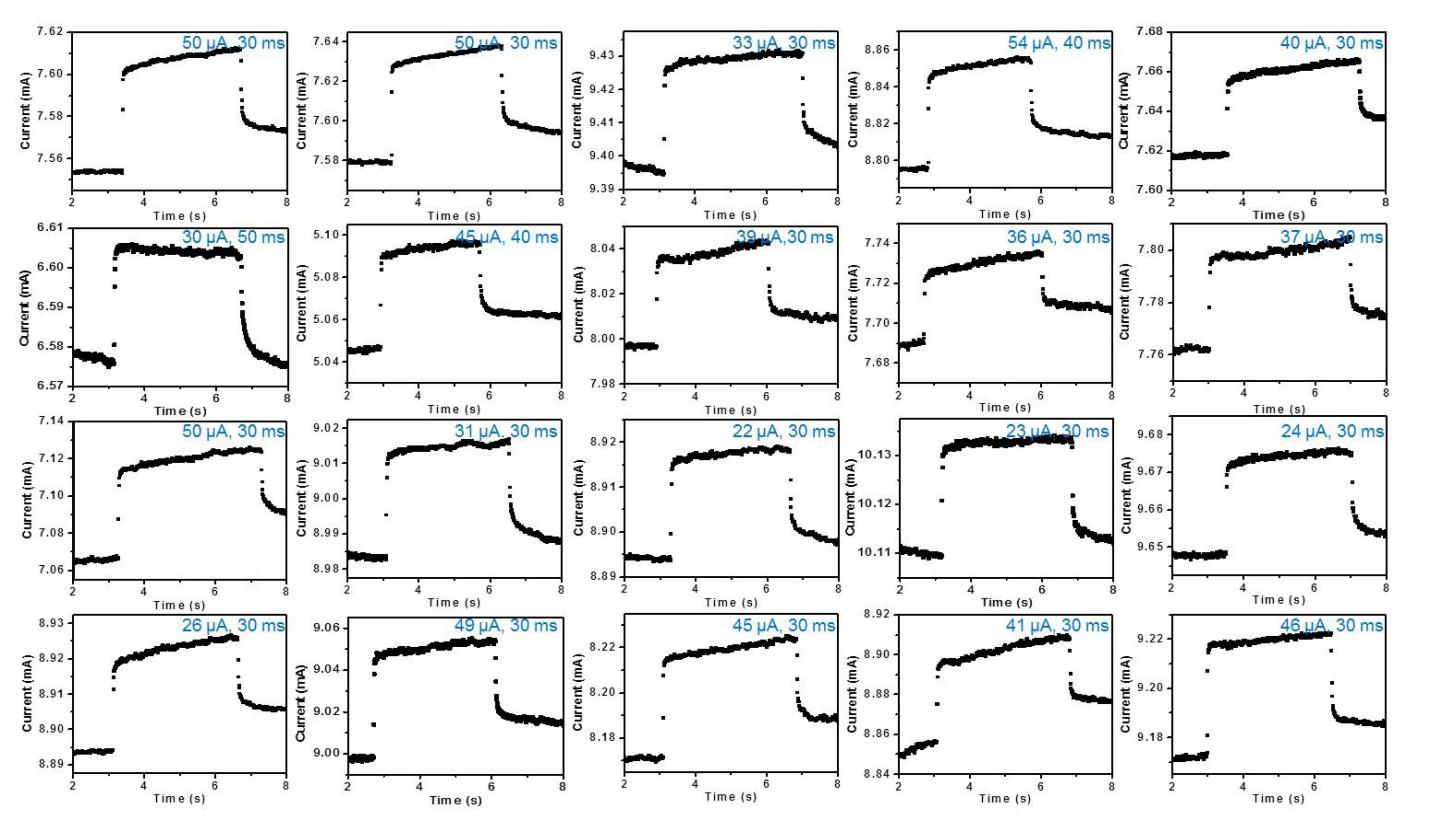


**Figure S10.** The time dependent response results of 20 devices at *V*_sd_ = 1 V with *V*_g_ = 0 V, by switching 460 nm light illumination with power of 1.4 mW/cm^2^ on and off.

**11. Time dependent response results of 20 graphene/CdSe film photodetectors**

For the reproducibility investigation of the photoconductive performance for the graphene/CdSe film hybrids, 20 photodetectors with channel length of 20 μm and width of 500 μm were fabricated by shadow mask. The time dependent response curves of 20 devices at *V*_sd_ = 1 V with *V*_g_ = 0 V are shown in Figure S11, with the values of photocurrent and response speed labelled in each figure.


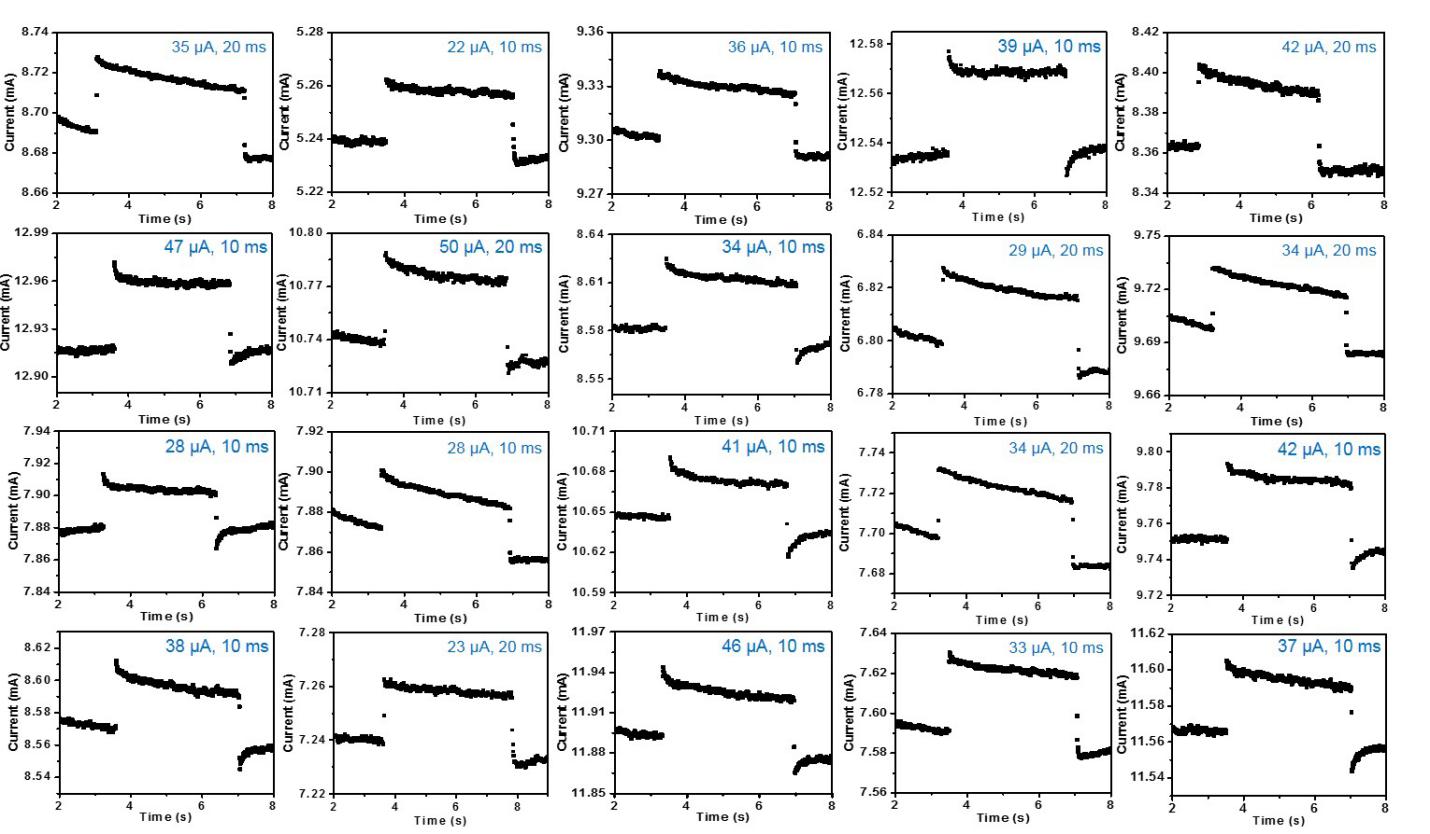


**Figure S11.** The time dependent response results of 20 devices at *V*_sd_ = 1 V with *V*_g_ = 0 V, by switching 620 nm light illumination with power of 1.4 mW/cm^2^ on and off.

**12. References**

1. Konstantatos, G. *et al*. Hybrid Graphene-quantum Dot Phototransistors with Ultrahigh Gain. *Nat Nanotechnol.* **7,** 363-368 (2012).

2. Wang, M. *et al*. CVD Growth of Large Area Smooth-edged Graphene Nanomesh by Nanosphere Lithography. *Sci Rep.* **3,** 1238 (2013).

3. Zhang, D.Y., Gan, L., Cao, Y., Wang, Q., Qi, L. M. & Guo, X. F. [Understanding Charge Transfer at PbS-Decorated Graphene Surfaces toward a Tunable Photosensor](http://apps.webofknowledge.com/full_record.do?product=UA&search_mode=GeneralSearch&qid=6&SID=3Cp5f7JtxnPFBNMnAzK&page=1&doc=1&cacheurlFromRightClick=no). *Adv Mater.* **24,** 2715-2720 (2012).
